# Frequency of Hypogonadism in Type 2 Diabetes Mellitus Patients with and without Coronary Artery Disease

**DOI:** 10.7759/cureus.6500

**Published:** 2019-12-29

**Authors:** Muhammad T Raza, Sabira Sharif, Zohaib Ahmad Khan, Sadaf Naz, Samsam Mushtaq, Amina Umer

**Affiliations:** 1 Medicine, Allama Iqbal Medical College/Jinnah Hospital, Lahore, PAK; 2 Internal Medicine, Shaikh Zayed Hospital, Lahore, PAK

**Keywords:** hypogonadism, frequency, coronary artery disease, diabetes mellitus, testosterone, deficiency

## Abstract

Introduction

Hypogonadism is characterized by clinical and biochemical evidence of testosterone deficiency. Low testosterone levels have been reported in patients with type 2 diabetes mellitus (T2DM), which can predispose to coronary artery disease (CAD). It has been proposed that diabetic men with proven CAD have lower androgen levels than patients with normal coronary arteriograms. We conducted this study with the objective to determine the frequency of hypogonadism in patients with diabetes mellitus and its relationship with CAD.

Materials and Methods

It was a comparative cross-sectional study conducted at a tertiary care hospital. We recruited a total of 108 patients, divided into two groups, 54 patients in each arm of the study. Group A comprised patients with CAD, whereas group B consisted of diabetic patients without CAD. Hypogonadism was defined on the basis of erectile dysfunction clinically and total testosterone levels biochemically. CAD was diagnosed on the basis of findings of coronary angiography. Fasting blood samples were drawn and evaluated for fasting plasma glucose, HbA1c, fasting lipid profile, thyroid-stimulating hormone (TSH), serum prolactin, blood urea, serum creatinine, liver function tests (LFT), total testosterone, luteinizing hormone (LH), and follicle‑stimulating hormone (FSH) levels. Hypogonadism among two study groups was compared using chi-square and serum testosterone level was compared using independent *t*-test with *p* < 0.05 considered as statistically significant.

Results

There were 108 subjects in the study with the mean age of 54.4 ± 4.29 (range: 22 to 68) years. The mean duration of T2DM was 12.6 ± 8.2 years. The mean BMI of patients with and without CAD was 25.7 ± 2.37 and 26.9 ± 4.21 kg/m^2^, respectively. There was no significant difference in waist circumference and obesity between both the groups (*p*-value > 0.05). Fasting plasma glucose and HbA1c in both groups were not significantly different. Testosterone levels and erectile dysfunction score were found lower in T2DM with CAD compared to T2DM patients without CAD, although this difference was not statistically significant (*p*-value: 0.051). The majority of the subjects with hypogonadism in both groups had a hypogonadotrophic hypogonadism (39/42, 92.9% versus 16/20, 80.0%). No statistically significant difference was seen in serum levels of LH and FSH between the study groups. The frequency of hypogonadism was found higher in the group with CAD (72.2%, 39/54) as compared with T2DM patients without CAD (37.03%, 20/54; *p*-value = 0.000).

Conclusion

Testosterone deficiency is a significant problem of males with T2DM. Patients with CAD have markedly low levels of testosterone as compared with patients without any CAD.

## Introduction

Hypogonadism is a clinical condition comprising both clinical and biochemical evidence of testosterone deficiency [1-3]. Males with type 2 diabetes mellitus (T2DM) have low testosterone levels in comparison to healthy individuals [4]. The reported prevalence of hypogonadism in different studies varies from 11.8% to 97.2% primarily due to differences in diabetic control in the study population [4-5].

There are multiple causes of low testosterone level in T2DM patients, which include decreased production of sex hormone-binding globulin (SHBG) by liver, which is a major carrier protein of testosterone in circulation [2]. Furthermore, visceral obesity among diabetics converts testosterone to estradiol by aromatase enzyme in adipose tissues. Low testosterone, in turn, leads to even more fat accumulation and augmentation of testosterone deficiency [6-7].

Framingham heart study suggests that low testosterone level increases the risk of coronary artery disease (CAD) [7]. Low testosterone level modifies cardiovascular risk factors particularly lipid profile, blood pressure, obesity, insulin resistance, inflammatory cytokines & fibrinogen levels, all of which are risk factors of CAD [8]. Testosterone also has vasoactive properties improving both systemic and coronary blood flow [7]. The effect of low testosterone on CAD remains significantly high even after adjustment of other confounding variables of CAD, including age, obesity, and dyslipidemia, which are also significant predictors of CAD in men with or without T2DM [9]. It has also been reported that diabetic men with proven CAD have lower androgen levels than patients with normal coronary arteriograms [10].

Although testosterone deficiency has proven to be a risk factor for CAD, this has received scant attention in the medical literature. So, we conducted this study with the objective to determine the frequency of hypogonadism in T2DM and its variation with regard to CAD.

## Materials and methods

It was a comparative cross-sectional study conducted at (redacted for peer review) from August 2018 to April 2019. This study was approved by the Ethical Review Board of (redacted for peer review). We recruited a total of 108 patients, divided into two groups, with 54 patients in each arm of the study. Group A comprised patients with CAD, whereas group B consisted of patients without CAD. Patients were labeled to have T2DM if they fulfilled one or more of the following criteria of the American Diabetic Association (ADA) [11-12].

1. Fasting blood sugar (FBS) ≥ 126mg/dl (7 mmol/L) after eight hours of fasting

2. HBA1c ≥ 6.5%

3. Random blood sugar (RBS) ≥ 200mg/dl (11.1mmol/L) with symptoms of hyperglycemia (polydipsia, polyuria, polyphagia, and weight loss).

4. Two-hour RBS ≥ 200 mg/dl (11.1mmol/L) on 75 g oral glucose tolerance test (OGTT)

Erectile dysfunction was labeled on the basis of the International Index of Erectile Dysfunction (IIEF) score. Patients with score less than 20 were diagnosed to be suffering from erectile dysfunction. Patients with total testosterone (TT) level below 3 ng/ml were diagnosed as having hypogonadism and were sub-categorized as mentioned below:

1. Hypogonadotropic hypogonadism (HH) was defined as TT level <3 ng/ml and luteinizing hormone (LH) values ≤10 IU/L.

2. Primary hypogonadism was defined as TT levels <3 ng/ml and LH levels >10 IU/L.

The following patients were excluded from the study:

1. Patients with type 1 diabetes mellitus

2. With a known history of hypogonadism, hyperprolactinemia/ prolactinoma, or panhypopituitarism, Klinefelter syndrome, cryptorchidism, varicocele, and any other disorder of androgen synthesis

3. Past history of any coronary event (myocardial infarction or unstable angina) or coronary procedure (coronary artery bypass or coronary angioplasty) in the previous four weeks

4. Liver disease (bilirubin ≥ 1.5 mg/dl), serum creatinine levels >1.5 mg/dl

5. Systemic disorders such as cancer, epilepsy, hypothyroidism, and evidence of any active infection

6. Alcoholics (≥500ml/week of alcohol for ≥ 6 months period)

7. Acute stress, anorexia nervosa, and malnutrition

8. History of mumps, radiation, alkylating agents, ketoconazole, glucocorticoid use, trauma

Group A - T2DM subjects with confirmed CAD by either one of prior coronary angiography or computed tomography (CT) angiography were included in this group. They were enrolled from the cardiology outpatients department under the supervision of the experienced cardiologist. Group B - T2DM subjects without CAD were enrolled from the Jinnah hospital Endocrinology department (JAIDE).

Patients were assured that all the information would be kept confidential and patient’s privacy would not be affected. After obtaining written informed consent from the patients, a biodata form was filled and detailed medical history and clinical examination were undertaken. Various parameters such as body mass index (BMI) and blood pressure (in sitting and standing postures) were recorded.

All the patients were given translated IIEF questionnaire, and erectile dysfunction was labeled as per operational definition. Fasting blood samples was drawn and sent to the hospital laboratory for analysis of plasma glucose, HbA1c, fasting lipid profile, thyroid-stimulating hormone (TSH), serum prolactin, blood urea, serum creatinine, liver function tests (LFT), total testosterone, luteinizing hormone, and follicle‑stimulating hormone (FSH). Effect modifiers such as age and BMI were addressed through the stratification of data.

The data were entered and analyzed through Statistical Package for Social Sciences (SPSS version 23.0, IBM Statistics, Chicago, IL, USA). Quantitative variables such as age, serum HbA1c, total testosterone, and IIEF score were calculated as mean ± SD. Categorical variables such as hypogonadism and presence of CAD were analyzed as frequencies and percentages. Hypogonadism among two groups was compared using chi-square test and serum testosterone level was compared using independent *t*-test with *p* < 0.05 considered as statistically significant.

## Results

There were 108 subjects in the study with a mean age of patients with CAD was 54.4 ± 4.29 (range: 22-68) years. 55.94 ± 4.14 (range: 48-68) years & without CAD 52.77 ± 4.44 (range: 22-60) years. The mean duration of T2DM was 12.6 ± 8.2 years. The mean BMI of patients with and without CAD was 25.7 ± 2.37 and 26.9 ± 4.21 kg/m^2^, respectively. Various clinical and biochemical parameters of the patients are presented in Table 1.

**Table 1:** Clinical and biochemical parameters of the study population CAD, coronary artery disease; HbA1c, glycated hemoglobin A1c

Parameters	Study Groups		P-value
	With CAD (Mean ± SD)	Without CAD (Mean ± SD)	
Age (years)	55.9 ± 4.1	52.8 ± 4.5	0.090
Body mass index (kg/m^2^)	25.7 ± 2.4	26.9 ± 4.2	0.060
Erectile dysfunction score	8.74± 2.8	10.8 ± 2.3	0.000
Testosterone level (ng/ml)	2.59 ± .5	2.89 ± .7	0.051
Luteinizing hormone (ng/ml)	8.19 ± 1.5	7.46 ± 2.7	0.090
Follicle-stimulating hormone (ng/ml)	7.37 ± 2.2	7.69 ± 2.4	0.467
Thyroid-stimulating hormone (ng/ml)	2.96 ± 1.2	2.81 ± .8	0.420
Prolactin levels (ng/ml)	8.65 ± 3.8	10.9 ± 1.9	0.000
Fasting blood sugar (mg/dl)	143 ± 16.9	149 ± 20.3	0.151
HBA1c (%)	10.6 ± 1.8	10.8 ± 1.9	0.74
Serum cholesterol (mg/dl)	205 ± 13.5	205 ± 19.3	0.822
Low Density Lipoprotein (mg/dl)	103 ± 10.5	108 ± 17.1	0.116
High density lipoprotein (mg/dl)	37.1 ± 3.2	37.7 ± 4.1	0.399
Serum triglyceride (mg/dl)	184 ± 19.9	166 ± 15.8	0.000
Blood urea (mg/dl)	17.7 ± 5.4	23.5 ± 5.0	0.000
Serum creatinine (mg/dl)	1.02 ± .1	1.05 ± .8	0.834
Systolic blood pressure (mmHg)	134 ± 12.4	129 ± 20.3	0.108
Diastolic blood pressure (mmHg)	83.9 ± 4.5	82.7 ± 7.9	0.354

Subjects in both groups were matched for age and BMIs. Table 2 shows the frequency of hypogonadism and erectile dysfunction in patients with and without CAD.

**Table 2:** Frequency of hypogonadism and erectile dysfunction in the study population

Parameter		Coronary artery disease		P-value
		Present	Absent	
Hypoganadism	Yes	39	20	0.000
		72.2%	37.0%	
	No	15	34	
		27.8%	63.0%	
Erectile dysfunction	Yes	40	21	0.000
		74.1%	38.9%	
	No	14	33	
		25.9%	61.1%	

There was no statistically significant difference in waist circumference and obesity between both groups (*p*-value > 0.05). Fasting plasma glucose and HbA1c levels in both groups were not statistically different.

Testosterone and IIEF scores were found lower in T2DM with CAD compared to T2DM patients without CAD, although this difference was not statistically significant (*p*-value: 0.051). The majority of the subjects with hypogonadism in both groups had HH (39/42, 92.9% versus 16/20, 80.0%). No statistically significant difference was seen in serum levels of LH and FSH between the study groups (*p*-value > 0.05).

The frequency of hypogonadism as indicated by low testosterone levels & IIEF score was found higher in Group A patients with CAD (72.2%, 39/54) compared to T2DM patients without CAD (37.03%, 20/54; *p*-value = 0.000). 

The relationship between hypogonadism and CAD in patients with different age groups and BMI is presented in Tables 3 and 4, respectively.

**Table 3:** Relationship between age and hypogonadism in patients with and without coronary artery disease CAD, coronary artery disease

Age groups	Presence or absence of hypogonadism		Study groups		Total	P-value
			With CAD	Without CAD		
<50 years	Hypogonadism	Yes	4	5	9	0.026
			100.0%	31.3%	45.0%	
		No	0	11	11	
			0.0%	68.8%	55.0%	
	Total		4	16	20	
			100.0%	100.0%	100.0%	
>50 years	Hypogonadism	Yes	35	15	50	0.004
			70.0%	39.5%	56.8%	
		No	15	23	38	
			30.0%	60.5%	43.2%	
	Total		50	38	88	
			100.0%	100.0%	100.0%	

**Table 4:** Relationship between hypogonadism and coronary artery disease in patients with different body mass indices BMI, body mass index; CAD, coronary artery disease

BMI (kg/m^2^)	Hypogonadism		Study group		P-value
			With CAD	Without CAD	
Under-weight (BMI < 18.5)	Hypogonadism	Yes	1	1	----
			100.0%	100.0%	
	Total		1	1	
			100.0%	100.0%	
Normal (BMI 18.5-24.9)	Hypogonadism	Yes	30	5	0.000
			75.0%	22.7%	
		No	10	17	
			25.0%	77.3%	
	Total		40	22	
			100.0%	100.0%	
Overweight (BMI 25 - 28.9)	Hypogonadism	Yes	7	11	0.311
			63.6%	47.8%	
		No	4	12	
			36.4%	52.2%	
	Total		11	23	
			100.0%	100.0%	
Obese (BMI > 30)	Hypogonadism	Yes	1	3	0.667
			50.0%	37.5%	
		No	1	5	
			50.0%	62.5%	
	Total		2	8	
			100.0%	100.0%	

## Discussion

In this study, we compared the frequency of testosterone profile of diabetic patients with and without CAD. We found a significantly higher prevalence of hypogonadism in diabetic subjects with CAD compared to diabetic subjects without CAD. Madhu* et al.* evaluated the prevalence of hypogonadism in T2DM and its relationship with CAD [1]. In their study, hypogonadism was found to be highest in T2DM patients with CAD (40%, 20/50) as compared to T2DM patients who did not have CAD (32%, 16/50; *p*-value: 0.53). The levels of total testosterone were significantly lower in patients with CAD than without CAD (3.55 ± 1.46 ng/ml vs. 4.73 ± 2.17 ng/ml; *P* = 0.005). They reported a positive correlation between hypogonadism and CAD (*r* = 0.177, *P* = 0.030). We also found similar results and observed greater frequency of CAD patients with hypogonadism and lower testosterone levels.

Hypogonadism occurs due to microvascular complications of diabetes and range from vasculopathy to neuropathy and endocrinal disorders. Certain studies have attributed psychogenic involvement to be the leading cause of erectile dysfunction in patients with hypogonadism. Previous studies attribute vasculopathy to be a more common culprit in causing erectile dysfunction and endocrine imbalance to be the leading cause of hypogonadism in diabetes mellitus.

Hypogonadotropic hypogonadism was observed in most of the patients of our study as reported earlier in another study [1]. Earlier reports have shown both higher and lower testosterone levels in hypogonadotropic male diabetic patients [11]. The proposed mechanism of this observation is a higher rate of conversion of serum testosterone to estradiol in patients with higher body fat content that occurs in patients with low testosterone levels [12]. The resultant suppression of gondatotrophin-releasing hormone (GnRH) axis leads to hypogonadotropic hypogonadism. On the contrary, primary hypogonadism is proposed to result from microvascular lesions in the testis, which damages the interstitium of the testis.

Deleterious cardiovascular effects of low testosterone levels have earlier been reported in various studies. A study showed a higher frequency of atherosclerosis and a greater presence of inflammatory markers in patients with low testosterone levels. An earlier report also showed increased thickness of the carotid intima-media layer in patients with low testosterone levels [13].

Whether testosterone deficiency should be corrected in patients with hypogonadism still remains a controversial topic. It has been proposed that testosterone replacement therapy can reduce all-cause cardiovascular mortality in patients with CAD. A recent retrospective study reported that testosterone therapy, in an attempt to normalize testosterone levels, resulted in significant lowering of the risk of myocardial infarction and stroke in the study population [14]. Different other studies have also shown that normalizing testosterone levels in hypogonadotropic males produce improvement in glycemic control by reducing insulin resistance. The study proposed that the effect of testosterone on cardiovascular mortality is due to its indirect effect by improving glycemic control and hence, reducing the prevalence of diabetic microvascular complications [15]. We recommend future studies to determine the effect of testosterone therapy on the cardiovascular system and see if it adds to an additional cardiovascular benefit to the treated patients [16-26].

## Conclusions

Testosterone deficiency is a significant problem of males with T2DM. Patients with CAD have markedly low levels of testosterone as compared with patients without any evidence of cardiovascular disease.
